# Evaluation of a Training for Health and Social Service Providers on Abortion Referral-Making

**DOI:** 10.1007/s10995-018-2570-6

**Published:** 2018-07-06

**Authors:** Jenny O’Donnell, Kelsey Holt, Kristin Nobel, Melanie Zurek

**Affiliations:** 1 Provide, PO Box 410164, Cambridge, MA 02141 USA; 2 Department of Family and Community Medicine, University of California, San Francisco, 1001 Potrero Ave, 2210, San Francisco, CA 94110 USA

**Keywords:** Unintended pregnancy, Abortion referrals, Coordination of care, Workforce development

## Abstract

*Purpose* Engaging trusted care providers and empowering them with information and skills about abortion is a critical opportunity to improve coordination of care for women seeking abortion, if and when these services are needed. *Description Provide*, a nonprofit that works in partnership with health and social service providers to build a health system that is equipped to respond to women’s health care needs around abortion, launched a referrals training program in 2013. To assess the effectiveness of this training program, we conducted an evaluation of satisfaction with training and the impact of the intervention on provider knowledge of safety of abortion, self-efficacy to provide abortion referrals, and intention to provide pregnancy options counseling and referrals in the future. *Assessment* Approximately 90% of participants were “very satisfied” with their training experience. Results show significant increase in intention to provide non-judgmental pregnancy options counseling and referrals for abortion care after participants went through training. Post-training, significantly more reported that they would present all pregnancy options without judgment or bias (94 vs. 82%, p < .0001), provide a referral for abortion care if needed (80 vs. 50%, p < .0001), and follow-up with the client (71 vs. 39%, p < .0001). Further, more also reported they would refer a client for prenatal care if the client requested it (78 vs. 67%, p < .0001). *Conclusion* Our results suggest that abortion referrals training hold potential to build the capacity of health and social service providers’ ability to meet client needs related to pregnancy and could be implemented at a larger scale.

## Significance

Engaging care providers and empowering them with information and skills about abortion is a critical opportunity to improve coordination of care for women seeking abortion, if and when these services are needed. A growing evidence base on pregnancy options counseling and abortion referral-making suggests deficiencies in clinicians’ practice.

The results document satisfaction with abortion referrals training and positive impact on provider knowledge of safety of abortion, self-efficacy to provide abortion referrals, and intention to provide pregnancy options counseling and referrals in the future. This suggests training is an effective method of improving provider knowledge and influencing their intended practice.

## Introduction

Access to unintended pregnancy care, including abortion services, is essential to the well-being of women and families (Institute of Medicine 1995; Upadhyay et al. 2015). In the United States, a large majority (90%) of counties have no abortion provider and most (59%) abortions are performed in specialized abortion clinics (Jones and Jerman 2017). Thus, many women seeking abortion must access care in a location apart from their usual source of health care. Engaging trusted care providers and empowering them with information and skills about abortion is a critical opportunity to improve coordination of care for women seeking abortion, if and when these services are needed. Abortion referral-making—defined here as a process of connecting a woman in need of abortion care with a facility that provides services—is a critical, yet often overlooked, component of access (Zurek et al. 2015). Difficulty locating a provider is associated with later gestational age at time of abortion (Drey et al. 2006; Waddington et al. 2015), which is in turn associated with more expensive clinical procedures and more major complications (Upadhyay et al. 2013).

A growing evidence base on abortion referral-making suggests deficiencies in clinicians’ practice (Dodge et al. 2012; French et al. 2015; Hebert et al. 2016; Holt et al. 2017; Homaifar et al. 2017). A recent national population-based survey of almost 800 primary care physicians (PCPs) revealed that 43% of family medicine and general internal medicine physicians reported seeing women seeking abortion (Holt et al. 2017). However, 38% of those who saw women seeking abortion and did not provide abortion themselves did not routinely provide any form of referral; this percentage varied by region. For example, it was almost 50% in the Southeast, with almost 20% of physicians further reporting routinely attempting to dissuade women from abortion. Another study found that, when prompted for a referral by a mystery client over the phone, only about half of staff working in reproductive health facilities that do not provide abortion provided a direct referral (defined as providing the name or telephone number of a facility that provided abortion services) (Dodge et al. 2012). A survey of publicly-funded family planning services in 16 states found that significantly fewer providers offer abortion referrals compared to adoption referrals (Hebert et al. 2016) and a survey in Nebraska of family medicine physicians and other clinicians found that providers were much less likely to say they would provide abortion referrals compared to other pregnancy related services (French et al. 2015).

Responding to these practice gaps, *Provide*, a nonprofit that works in partnership with health and social service providers to build a health system that is equipped to respond to women’s health care needs around abortion, launched a referrals training program in 2013. Because social service providers, such as social workers, case managers, or counselors, also play an important role in connecting women to unintended pregnancy care, the training targets social service providers in addition to clinicians. The training is also available for clinical and social service administrators and support staff, who may be under-engaged in creating the expectations around and conditions for referral-making (Zurek et al. 2015). To assess the effectiveness of this training program, we conducted an evaluation of satisfaction with training and the impact of the intervention on health and social service provider knowledge of safety of abortion, self-efficacy to provide abortion referrals, and intention to provide pregnancy options counseling and referrals in the future.

## Materials and Methods

### Training Program and Participants

In 2013, *Provide* worked with curriculum development experts to develop *Referrals for Unintended Pregnancy: A Curriculum for Health and Social Service Providers*. This curriculum offers professional development for health and social service providers on how to give accurate, informed, and non-judgmental referrals for abortion care. Because comprehensive skills and information about abortion care are often left out of all-options pregnancy training for many professionals, this training and associated technical assistance offers tools and resources to equip workers in these sometimes difficult discussions with clients.

The target audience for this training includes healthcare providers (e.g. physicians, nurse practitioners, other advance practice clinicians, and registered nurses), social service providers (e.g. social workers, case managers, counselors), support staff (e.g. front desk staff, hotline volunteers), and administrators (e.g. nurse managers, site directors). The training modules include: (1) Why Refer; (2) How to Help; (3) What: Special Topics in Reproductive Health Referral-Making; (4) Who: Meeting Specialized Service Providers; (5) Respect and Continuity of Care; and (6) Options Counseling (training materials are available to the public upon request, along with technical assistance supporting their use).

All trainings take place in person. The duration of training sessions is variable, with training content tailored to the needs of the site. Modules are offered in one continuous block or over multiple sessions. However, in order to be defined as a training (and included in the following analysis), core training modules (one through three) must have been offered. From 2014 to 2016, the training was also available in Spanish for those sites that provided services primarily in that language across all eligible states. State-based training teams offer on-going follow-up technical support to integrate training content into work with clients and patients. The teams also offer regularly updated resource lists so that health and social service providers have the information needed to make effective abortion referrals. Training and technical assistance are provided with no cost to the service delivery site or the trainees.

Over the course of 4 years (2013–2016), trainers across seven Southeastern states conducted 271 trainings and served 2929 participants. Eligible trainings sites included service delivery locations offering ‘safety net’ family planning services, domestic violence/sexual assault services, HIV services, substance abuse services, and publicly funded primary care services. The categories of eligible sites were not mutually exclusive (for example, a site may offer both family planning and HIV services). The target service delivery systems in each state varied, with *Provide* identifying sites within a set of service delivery systems through a strategic mapping process. Eligibility criteria for sites included being known providers of at least one of the services listed above (i.e. services are advertised), being direct service providers, serving women of reproductive age, and being publicly funded in some way. Once a system was selected, the state team conducted outreach to all identified sites, creating demand for participation in this free training by articulating how the site’s workforce and clients/patients could potentially benefit from the additional capacity-building opportunity offered.

Sites determined which staff members attended the training, with an emphasis on individuals who, in their role, might encounter a client with an unintended pregnancy and be in a position to provide counseling or referral. The structure of participation varied by sites; for some sites, attendance was mandatory for all staff, while others invited participation on a voluntary basis. While conditions of attendance were not recorded systematically in program records, two large organizations (representing 17% of all participants) are known to have required attendance.

### Measures

Anonymous surveys were distributed to participants prior to and immediately following each training. The measures used in the survey instrument were developed specifically for this program evaluation, placing a high priority on constructions that were highly feasible to administer and most meaningful for ongoing refinement of program implementation. Completed surveys were matched using a participant-generated ID code.

Post-workshop surveys assessed participant satisfaction with the following elements of the training: information provided by presenter, teaching methods and activities, and the way topics were addressed. Response options included: not satisfied, somewhat satisfied, and very satisfied.

Participants’ knowledge about abortion was assessed using the following survey statement: “Abortion in a clinic is a medically safe procedure.” Participants’ self-efficacy in providing options counseling and abortion referrals was assessed by asking participants about the extent to which they agreed with the following statements: “I have the skills and information I need to effectively counsel a client with an unintended pregnancy on all her options,” “I have the skills and information I need to effectively refer a client with an unintended pregnancy for pregnancy termination if she requests it,” and “In my workplace, I can provide counseling or referrals for any reproductive health service without fear of judgment by coworkers.” Response options for all were: strongly agree, agree, disagree, and strongly disagree.

Surveys assessed abortion referral-making behavior pre-workshop and intentions related to referral-making behavior post-workshop by asking how they usually respond (pre) or intend to respond (post) to clients who have an unintended pregnancy and are considering pregnancy termination. Respondents were asked to select all of the following pre-generated response options that applied: “refer to another colleague who is more comfortable/better prepared to handle these cases,” “refer to a ‘crisis pregnancy center’ or similar organization that will encourage continuing the pregnancy,” “encourage the client to continue her pregnancy,” “present all pregnancy options without judgment or bias,” “provide a referral for abortion care if the client requests it,” “follow-up with the client afterwards to determine whether or not she had a positive outcome,” and “N/A.” “Provide a referral for prenatal care if the client requests it” was also listed for comparison purposes.

### Analysis

Descriptive statistics were calculated for all outcome variables related to knowledge, self-efficacy, and behaviors. In order to examine the relationship of several independent variables with the primary outcome (intention to begin providing abortion referrals among those not already providing), we conducted a series of univariate logistic regression analyses and a multivariate logistic regression analysis. Due to the exploratory nature of the study, we included all available predictor variables hypothesized to influence the primary outcome in the multivariate model as covariates: system, professional role (with similar roles combined to reduce the number of categories), whether they had previously counseled women considering abortion, and satisfaction with training (dichotomized into % “Very Satisfied” with the way topics were approached and % Not “Very Satisfied”).

Analysis of de-identified evaluation data from *Provide’s* training program received an exempt determination from the Harvard T.H. Chan School of Public Health institutional review board.

## Results

### Response Rate

Of 2929 trainees, 2704 (92%) completed a survey prior to training and 2620 (89%) completed a survey after the training; 2489 (85%) had a matching survey pair. Sample sizes vary by outcome due to missing data and introduction of new questions in later years of the training evaluation.

### Participant Characteristics

Characteristics of participants who filled out at least one survey are described in Table 1. Participants represented a variety of professional roles, including administrators and those involved in direct patient care. Trainings took place in seven Southeastern states in a variety of sectors of the health and social services system. Four percent of trainees participated in Spanish language training.

**Table 1:** Participant characteristics (N = 2835)

	n	%
Professional role (question introduced 2015; n = 1840)		
Administrative role	343	19
Client educator/client advocate	294	16
Counselor/case worker/case manager	429	23
Physician	34	2
Nurse practitioner/certified nurse midwife/physician assistant	42	2
Medical assistant/registered nurse	228	12
Social worker	269	15
Other	201	11
State		
AL	155	5
KY	507	18
NC	404	14
OK	329	12
SC	842	30
TN	232	8
WV	366	13
Health or social service system		
Domestic violence/sexual assault	1057	37
Family planning	558	20
HIV	243	9
Latino health/advocacy	129	4
Native American/Tribal Organization	86	3
Medical/nursing school	228	7
Substance abuse	328	8
Other	206	12
Training conducted in Spanish		
No	2716	96
Yes	119	4
Year of training		
2013	96	3
2014	749	26
2015	726	36
2016	1264	45

131 pre only, 215 post only, 2489 matched pairs

Respondents who did not complete a matching Pre and Post survey were not included in analyses that compared Pre with Post responses. The percentage of respondents with paired surveys varied significantly according to state, system, and year (p < .0001 for these variables). The percentage of participants with paired surveys was lowest among participants from Native American/Tribal organizations (73%), participants from Alabama (79%), and participants trained in the program’s first year (54%). The percentage of respondents with matched surveys did not vary significantly by professional role or whether they received the training in Spanish (results not shown).

### Satisfaction with Training

Analysis of participant satisfaction included responses from all participants who responded to satisfaction questions on their Post-training survey. Less than 1% of participants indicated that they were “not satisfied” with the way topics were addressed, the teaching methods and activities, or the information provided by presenters (Table 2). On all three indicators, approximately 90% of participants were “very satisfied” with their training experience.

**Table 2:** Participant satisfaction

Satisfaction	Not satisfied n (%)	Somewhat satisfied n (%)	Very satisfied n (%)
The way topics were addressed (n = 2585)	21 (0.8%)	237 (9.2%)	2327 (90.0%)
The teaching methods and activities (n = 2594)	12 (0.5%)	249 (9.6%)	2333 (89.9%)
The information provided by the presenters (n = 2598)	19 (0.7%)	225 (8.7%)	2354 (90.6%)

### Abortion Knowledge and Referral Self-efficacy

After the training, a large majority (96%) agreed or strongly agreed abortion in a clinic is a medically safe procedure, up from 77% pre-training (p < .0001) (Table 3). A large majority (94%) agreed or strongly agreed that they could provide reproductive health referrals without judgment by coworkers, up from 84% pre-training (p < .0001). A large majority believed that they had the skills and information necessary to effectively refer a client for pregnancy termination (95%) and counsel a client with an unintended pregnancy on all her options (94%) after the training, up from 46 and 51%, respectively, pre-training (p < .0001).

**Table 3:** Abortion safety knowledge and referral self-efficacy

	Strongly disagree n (%)	Disagree n (%)	Agree n (%)	Strongly agree n (%)
Abortion in a clinic is a medically safe procedure (n = 2307)				
Pre	120 (5%)	420 (18%)	1139 (49%)	628 (27%)
Post*	24(1%)	67 (3%)	821 (36%)	1395 (60%)
I can provide reproductive health referrals without fear of judgment by coworkers (n = 2312)				
Pre	65 (3%)	310 (13%)	1222 (53%)	715 (31%)
Post*	24 (1%)	125 (5%)	1048 (45%)	1115 (48%)
I have the skills and information I need to effectively refer a client for pregnancy termination if she requests it (n = 2412)				
Pre	226 (9%)	1078 (45%)	806 (33%)	302 (13%)
Post*	22 (1%)	91 (4%)	1129 (47%)	1170 (49%)
I have the skills and information I need to effectively counsel a client with unintended pregnancy on all her options (n = 2417)				
Pre	167 (7%)	1008 (42%)	926 (38%)	315 (13%)
Post*	15 (1%)	123 (5%)	1248 (52%)	1030 (43%)

*p < .0001 in Chi-Square analysis comparing agree/strongly agree to disagree/strongly disagree

**Fig. 1:**
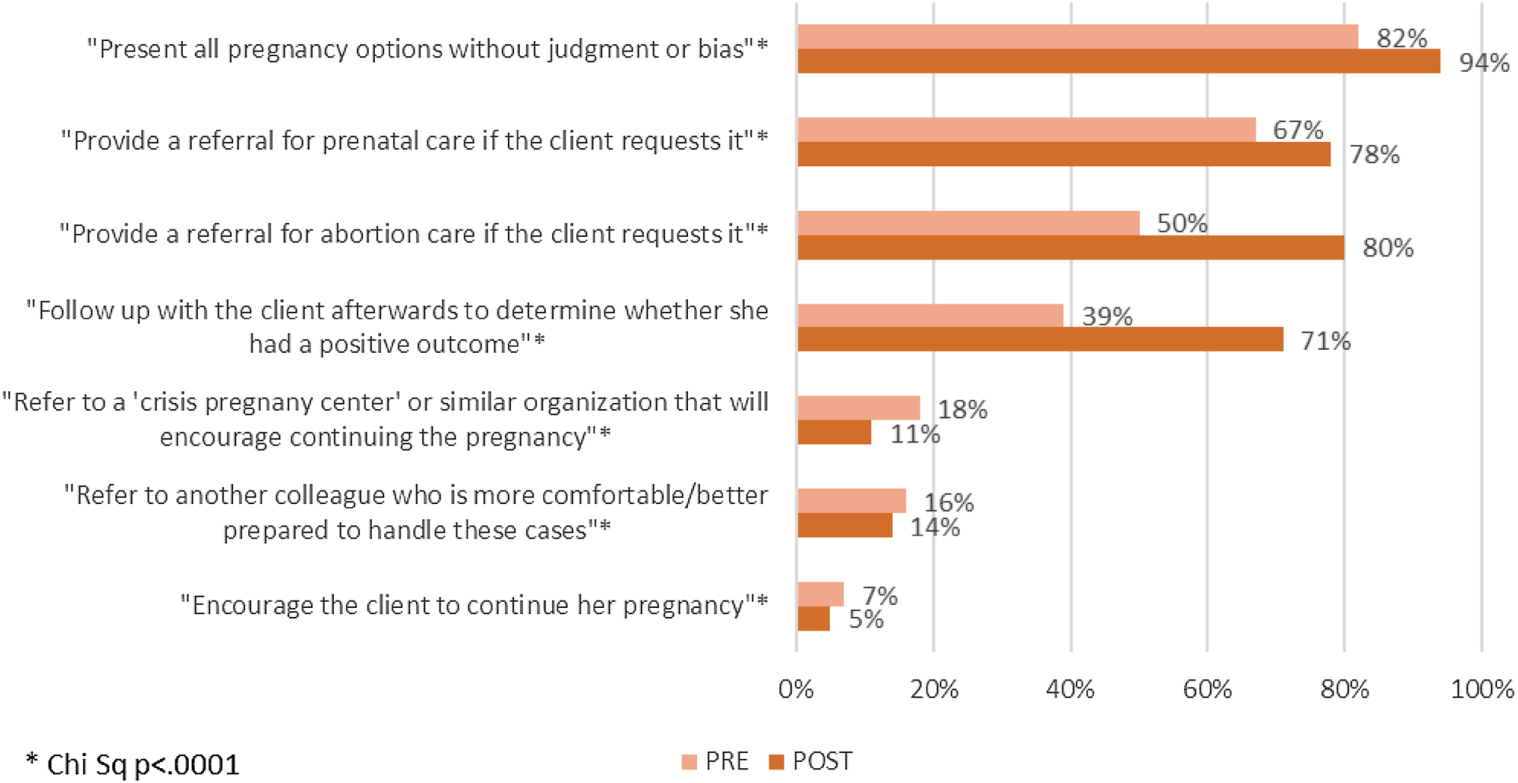
Counseling behavior for unintended pregnancy (reported past practice and future practice intentions) (n = 1376). Of the 2267 survey respondents who responded to behavior questions at both Pre and Post, 891 (39%) were excluded from a comparison of past practice with future intentions because they indicated at Pre that they had not yet had any clients with an unintended pregnancy who considered termination and thus were not queried about their current behaviors

Among participants who had discussed abortion as an option with women prior to the training (N = 1376 of 2267 total respondents to this question), several significant shifts were observed in their reported intentions for how they would handle those clients after participating in the training (Fig. 1). Significantly fewer reported that they would refer to a ‘crisis pregnancy center’ or similar organization that will encourage continuing the pregnancy (11 vs. 18%, p < .0001) or encourage the client to continue the pregnancy (5 vs. 7%, p < .0001). Significantly more reported that they would present all pregnancy options without judgment or bias (94 vs. 82%, p < .0001) and provide a referral for abortion care if needed (80 vs. 50%, p < .0001). Further, more reported that they would refer a client for prenatal care if the client requested it (78 vs. 67%, p < .0001) and follow-up with the client (71 vs. 39%, p < .0001).

Among all participants with a matching Pre–Post survey pair, 32% (n = 720) reported already providing abortion referrals compared to the 68% (n = 1537) who had not, either because they had not previously counseled a woman considering abortion (55%, n = 850) or they had, but had not offered a referral (45%, n = 687). Although only 32% of all participants had previously referred for abortion, 78% of all participants indicated in their Post-training survey that they intended to refer for abortion in future practice (significant difference between Pre-training practice and Post-training intention for matched pairs, p < .0001).

In univariate analyses, high satisfaction with training, select professional roles (Client Educator/Advocate, Social Worker, and Other, compared to Counselor/Case Worker/Case Manager), and several types of service delivery (domestic violence/sexual assault, and HIV, compared to Family Planning) were significantly correlated with an intended change in practice to provide referrals, among participants who had not previously provided abortion referrals (Table 4). In multivariate analysis, only the Domestic Violence/Sexual Assault system and the Social Worker role remained significantly associated with intended practice change, while satisfaction remained significant. Those who had not yet had any clients with unintended pregnancy were also more likely to report that they will begin providing referrals.

**Table 4:** Intention to begin providing abortion referrals: odds ratios (ors) from univariate and multivariate logistic regression

	Univariate OR (95% CI)	Multivariate OR (95% CI)
High satisfaction (“very satisfied”) with training	2.33 (1.67–3.25)****	2.47 (1.57–3.89)****
No prior counseling of clients considering abortion	1.63 (1.31–2.03)****	1.58 (1.18–2.11)**
Professional role		
Counselor/case worker/case manager (reference)		
Administrative role	0.92 (0.61–1.39)	0.97 (0.63–1.52)
Client educator/client advocate	1.72 (1.14–2.62)**	1.47 (0.94–2.30)
Medical provider—physician	1.53 (0.51–5.66)	0.95 (0.16–5.97)
Medical provider—CNM, PA, NP	1.00 (0.34–3.35)	1.15 (0.34–4.17)
Medical provider—MA or RN	1.00 (0.64–1.60)	1.24 (0.73–2.13)
Social worker	1.76 (1.13–2.78)*	1.85 (1.17–2.98)**
Other	4.26 (2.48–7.73)****	1.23 (0.13–7.60)
Health or social service system		
Family planning (reference)		
Substance abuse	1.22 (0.83–1.80)	1.26 (0.72–2.22)
Medical or nursing school	4.67 (2.79–8.17)****	5.05 (0.90–46.8)
Native American/Tribal	0.59 (0.31–1.11)	0.86 (0.27–2.83)
Latino health	0.91 (0.54–1.56)	2.06 (0.89–5.12)
HIV	1.61 (1.03–2.55)*	1.46 (0.80–2.69)
Domestic violence/sexual assault	1.83 (1.34–2.48)****	1.81 (1.12–2.92)*
Other	0.92 (0.58–1.46)	1.20 (0.63–2.32)

Multivariable analysis was run for 1044 participants who did not report referral-making at baseline and who had complete information for predictor variables; the N for univariable analyses ranged from 1053 to 1537 depending on the amount of missing data in the predictor

*p < .05

**p < .01

***p < .001

****p < .0001

## Discussion

Engaging trusted health and social service providers and empowering them with information and skills about abortion is a critical opportunity to improve coordination of care for women seeking abortion, if and when these services are needed. However, existing literature reveals an absence of evidence-based training available to support health and social service providers in their professional role around this aspect of care. A scan of the available training options (evidence-based or other) reveals that *Provide’s* training is unique in its attention to referrals, a component of quality pregnancy options counseling (Simmonds and Likis 2005) that is under-explored. In addition, the training is unique in its depth around specific and accurate abortion-related information, which is critical to responding to women’s needs related to unintended pregnancy but is often absent from existing resources. Finally, many existing training resources are not available via in-person delivery and are not aimed at a variety of health and social service professionals in a range of settings.

The results of this evaluation suggest that *Provide’s* referrals training is a promising practice, supported by the significant increase in immediate-term intention to provide these services after participants went through training. A positive effect is also seen relating to client follow-up and, though not an intended outcome of the training, referrals to prenatal care. Increases in associated measures relating to perceptions regarding safety, participant knowledge and skill, and co-worker support point to the possible mechanisms by which the training leads to intended behavior change. The low baseline (particularly in comparison to other measures) and substantial increase in provider belief that they have the information and skills they need to provide options counseling and referral is especially notable, and suggests substantial opportunity for improvement in professional education and training. High satisfaction rates suggest that this approach to abortion referrals training is acceptable to health and social service providers.

While increases in all measures were seen throughout, findings from multivariate analysis offer information on which sub-groups most benefited from the training. Being a social worker, compared to a counselor, or working in Domestic Violence/Sexual Assault, compared to Family Planning, were both associated with significantly higher rates of intending to initiate abortion referrals, after controlling for other personal and practice characteristics. Similarly, participants who had not previously counseled clients considering abortion were more likely to report an intention to provide referrals in the future. These differences point to the possibility that intended behavior change is greatest where the need for support around unintended pregnancy is easily recognized (such as in the arena of Sexual Assault and Domestic Violence), but the information and skills presented are new. Among participants who had previously counseled clients considering abortion, decreased intentions regarding behaviors that discourage abortion show additional evidence of a potential benefit, if a small effect size.

While the results of this evaluation show promise as to the feasibility and immediate impact of the referrals training, they are preliminary and have limitations. The immediate impact on provider attitudes and intended behavior does not provide evidence of changes in the long-term. Further, the measures used in the trainee survey instrument were developed specifically for this program evaluation, placing a high priority on constructions that were highly feasible to administer and most meaningful for ongoing refinement of program implementation. However, these measures are not validated and the underlying constructs of interest were explored via a limited number of items. This limitation is most relevant for findings related to complex constructs (e.g. satisfaction, attitudes), and present less concern for findings related to more straightforward constructs (e.g. intention). Further research is needed to determine if immediate shifts translate into long-term shifts, and the extent to which such trainings will impact actual referral behavior and client outcomes is yet to be determined. Additionally, the results demonstrate associations, but not causality; the lack of a control group limits our ability to determine whether there is a causal link between the training and provider knowledge, self-efficacy, and intended behavior, or whether other factors account for the change. In addition to further exploration of the impact of the trainings, a growing emphasis on continuity/coordination of care within health care delivery invites consideration of the role of abortion referral making in relation to other intersecting health and social needs.

## Implications for Practice

The available evidence demonstrates that *Provide’s* referrals training is an effective method of improving provider knowledge and influencing their intention to change options counseling and abortion referral practices. This is of national clinical significance given that inadequate referral procedures are often the limiting factors in obtaining access to abortion in the U.S. (Zurek et al. 2015). Our results suggest that abortion referrals training holds potential to build the capacity of health and social service providers’ ability to meet client needs related to unintended pregnancy and could be implemented and tested at a larger scale.
